# Relevance of a TCGA-derived Glioblastoma Subtype Gene-Classifier among Patient Populations

**DOI:** 10.1038/s41598-019-43173-y

**Published:** 2019-05-15

**Authors:** Wan-Yee Teo, Karthik Sekar, Pratap Seshachalam, Jianhe Shen, Wing-Yuk Chow, Ching C. Lau, HeeKyoung Yang, Junseong Park, Seok-Gu Kang, Xiaonan Li, Do-Hyun Nam, Kam M. Hui

**Affiliations:** 1 Humphrey Oei Institute of Cancer Research, Department of Cellular & Molecular Research, National Cancer Center Singapore, Singapore, Singapore; 2 KK Women’s & Children’s Hospital, Singapore, Singapore; 3 Singhealth Duke-NUS Academic Medical Center, Singapore, Singapore; 4 Cancer & Stem Cell Biology Program, Duke-NUS Medical School, Singapore, Singapore; 5 Department of Pediatrics, Division of Hematology-Oncology, Texas Children’s Cancer Center, Houston, Texas, USA; 6 Baylor College of Medicine, Houston, Texas USA; 7 Dan L. Duncan Cancer Center, Houston, Texas, USA; 8 Institute of Molecular & Cell Biology, A*STAR, Singapore, Singapore; 9 Department of Neurosurgery, Samsung Medical Center, Sungkyunkwan University School of Medicine, Seoul, Republic of Korea; 10 Department of Neurosurgery, Brain Tumor Center, Severance Hospital, Yonsei University College of Medicine, Seoul, Republic of Korea; 11 Ann & Robert H. Lurie Children’s Hospital of Chicago, Chicago, Illinois USA; 12 Northwestern University Feinberg School of Medicine, Chicago, Illinois USA; 13 Department of Biochemistry, Yong Loo Lin School of Medicine, National University of Singapore, Singapore, Singapore

**Keywords:** Cancer genomics, Oncology

## Abstract

Glioblastoma multiforme (GBM), a deadly cancer, is the most lethal and common malignant brain tumor, and the leading cause of death in adult brain tumors. While genomic data continues to rocket, clinical application and translation to patient care are lagging behind. Big data now deposited in the TCGA network offers a window to generate novel clinical hypotheses. We hypothesized that a TCGA-derived gene-classifier can be applied across different gene profiling platforms and population groups. This gene-classifier validated three robust GBM-subtypes across six different platforms, among Caucasian, Korean and Chinese populations: Three Caucasian-predominant TCGA-cohorts (Affymetrix U133A = 548, Agilent Custom-Array = 588, RNA-seq = 168), and three Asian-cohorts (Affymetrix Human Gene 1.0ST-Array = 61, Illumina = 52, Agilent 4 × 44 K = 60). To understand subtype-relevance in patient therapy, we investigated retrospective TCGA patient clinical sets. Subtype-specific patient survival outcome was similarly poor and reflected the net result of a mixture of treatment regimens with/without surgical resection. As a proof-of-concept, in subtype-specific patient-derived orthotopic xenograft (PDOX) mice, Classical-subtype demonstrated no survival difference comparing radiation-therapy versus temozolomide monotherapies. Though preliminary, a PDOX model of Proneural/Neural-subtype demonstrated significantly improved survival with temozolomide compared to radiation-therapy. A larger scale study using this gene-classifier may be useful in clinical outcome prediction and patient selection for trials based on subtyping.

## Introduction

Glioblastoma (GBM), a deadly brain cancer, is the most lethal and common malignant brain tumor and the leading cause of death in adult brain tumors. Despite the advances in genomics and molecular classification^1–8^, the survival of GBM patients has not improved over the last decade^9–11^. The median survival of GBM patients is less than 16 months despite a multitude of therapies^9–11^. Standard of care is radiation therapy and temozolomide, which gives the best 2-year overall survival of about 25%^9–11^. While genomic data continues to rocket, clinical application and translation to patient care are lagging behind. Big data now deposited in The Cancer Genome Atlas (TCGA) network offers a window to generate novel clinical hypotheses. One overarching theme is how *genomics* can be applied to derive clinically relevant information to improve *therapy* for patients. We hypothesized that a TCGA-derived gene-classifier can be applied across different gene profiling platforms and population groups. Using an enhanced gene expression data set from Caucasian-predominant TCGA-cohorts, we derived a subtype gene-classifier and validated our subtype-prediction model on six different platforms over a decade (Caucasian-predominant cohorts: Affymetrix U133A = 548, Agilent Custom Array = 588 and RNA-seq = 168 deposited in TCGA, and additional three Asian-cohorts Affymetrix Human Gene 1.0 ST array = 61; Illumina HumanHT-12 v4 Expression BeadChip = 52; Agilent 4 × 44 K Whole Genome Oligo Microarray = 60). This provides institutions profiling patient tumors on various platforms with a model to predict GBM-subtypes on different patient populations (Caucasian, Korean and Chinese patients). In a therapeutic context, patient survival curves reflect a net result of a mixture of different treatment regimens, with or without surgical resection of varying extent. If we are able to dissect the knowledge of a particular GBM-subtype which is more temozolomide-responsive or radiotherapy-responsive, this will provide a core treatment stem for testing novel agents in combination, targeting at a specific GBM-subtype. As a proof-of-concept, we postulated that survival differed by subtypes in context of GBM-relevant therapies, and any difference in treatment response will be clearer if we study the individual effects with monotherapy arms of the two most common treatment modalities in GBM, radiation therapy versus temozolomide, in a panel of patient-derived orthotopic xenograft mice (PDOX) without any surgical resection of tumor (lessening tumor burden) or a mixture of therapies confounding the survival data. Our findings from this study, if done on a larger scale, may be useful in clinical outcome prediction and patient selection for trials based on subtyping. Our gene-classifier was validated on six different gene profiling platforms and relevant among populations of Caucasian, Korean and Chinese patients.

## Results

### Derivation of a subtype gene-classifier applicable for glioblastoma tumors profiled on multiple platforms among Caucasian, Korean and Chinese populations

**Figure 1:**
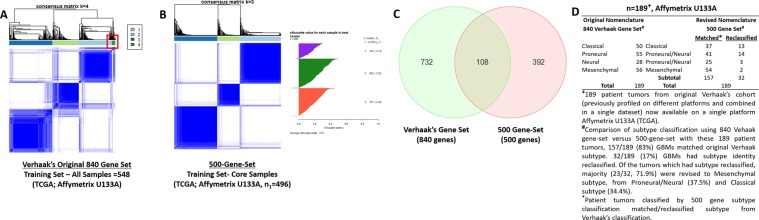
Derivation of a glioblastoma-subtype gene-classifier through three different platforms. Caucasian-predominant cohorts, TCGA: All samples - Affymetrix U133A = 548 (training set), Agilent = 588, RNA-Seq = 168. (**A**) Consensus clustering matrix for k = 4 of 548 glioblastoma (GBM) samples (training set) using Verhaak’s^1^ 840-gene-set revealed a small, unstable 4^th^ cluster (*red box*), suggesting the previous 840-gene-set comprised of insufficient genes to classify current larger TCGA-cohort (Supplemental 1). (**B**) Consensus clustering matrix for k = 3 of same training set using 500 gene-classifier and silhouette plot identified 496 core samples (n_1_ = 496), Supplemental 2 (k = 2 to k = 10). 500 gene-classifier comprised of top 500 differentially expressed genes between patient tumors (548 samples) and normal brain tissues (10 samples) curated through rigorous interrogation using 1500, 1000 and 500-gene-sets (Supplemental 2) in systematic genomic simulations derived from training set core samples. (**C**) Only 108 genes overlapped between 500 gene-classifier and Verhaak’s^1^ original 840 genes. (**D**) Comparison of subtype nomenclature using Vehaak’s^1^ 840-gene-set versus 500-gene-set in 189 patient tumors (original Verhaak’s^1^ cohort) revealed 157/189 (83%) GBM tumors matched original Verhaak^1^ subtype nomenclature. Importantly, 32/189 (17%) patient GBM tumors had subtype nomenclature *revised*.

**Figure 2:**
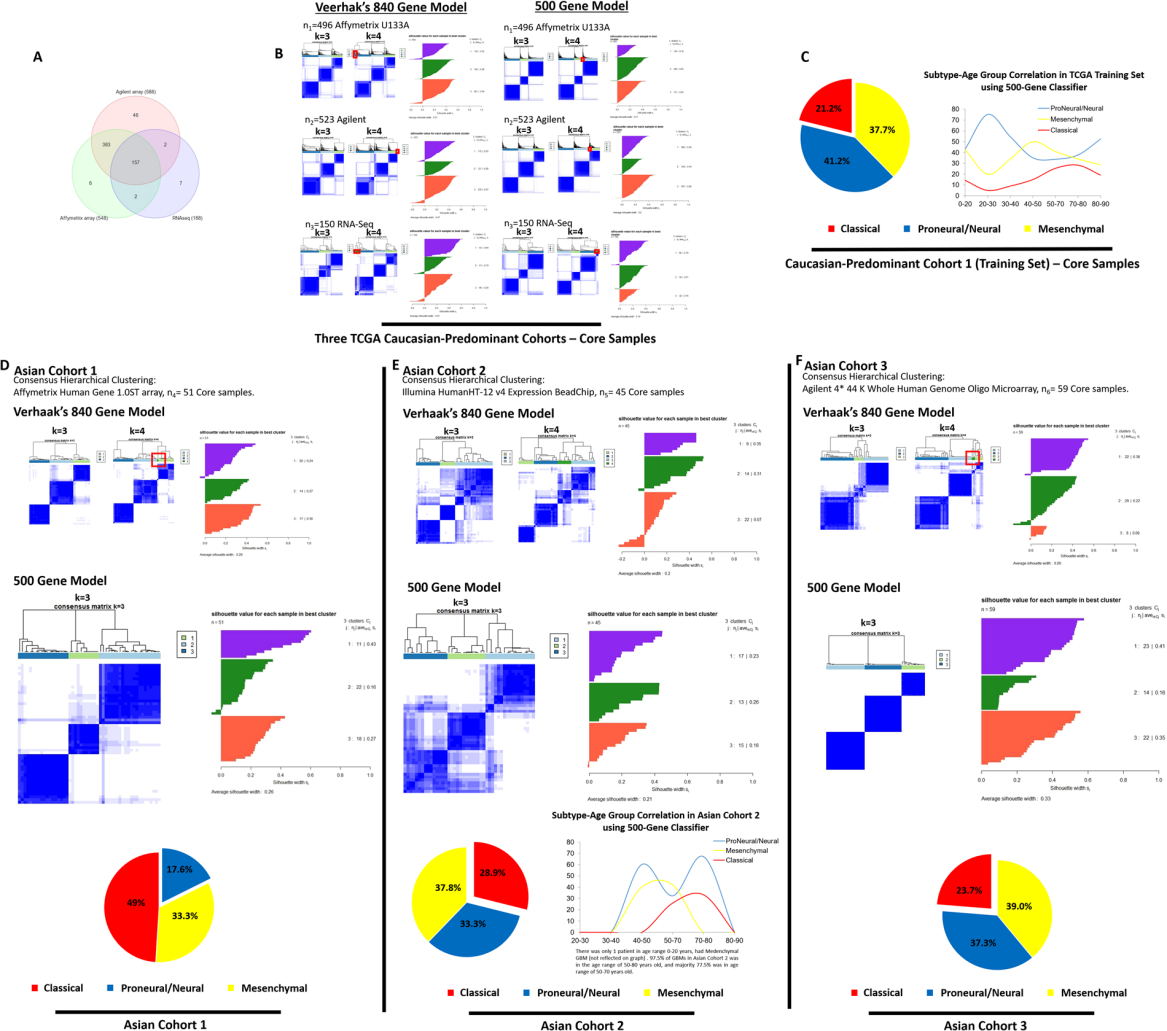
Comparison of Vehaak’s^1^ 840 gene model with 500 gene model and cross-ethnic application of 500 gene-classifier illustrating subtype predominance among patient populations of Caucasians, Koreans and Chinese. (**A**) Sample sizes of all three TCGA-cohorts: 157 patient tumors overlapped between three cohorts. (**B**) Comparison of consensus hierarchical clustering plots using Vehaak’s^1^ 840-gene-set versus 500 gene-classifier (k = 3, k = 4) across all TCGA-cohorts: Core samples n_1_ = 496 Affymetrix U133A, n_2_ = 523 Agilent, n_3_ = 150 RNA-Seq. Vehaak’s^1^ 840-gene-set produced less distinct clusters compared to 500-gene-set (k = 3). A small, unstable 4^th^ cluster (*red box*) emerged with k = 4. (**C**) Subtype distribution in 430 Caucasian GBM patients with ethnicity data available in training set (n_1_ = 496). (**D**–**F**) Comparison of consensus hierarchical clustering plots using Vehaak’s^1^ 840-gene-set versus 500 gene-classifier (k = 3, k = 4) across Asian-cohorts: Core samples: n_4_ = 51 Affymetrix Human Gene 1.0 ST array, n_5_ = 45 Illumina HumanHT-12 v4 Expression BeadChip, n_6_ = 59 Agilent 4 × 44 K Whole Genome Oligo Microarray. 500 gene-classifier recapitulated three GBM subtypes and produced more distinct clusters compared to Vehaak’s^1^ 840-gene-set in two Asian-cohorts of Korean descent and one Asian-cohort of Chinese descent. Classical subtype (49%) was the predominant subtype in Asian Cohort 1. Classical subtype was less common in Asian Cohort 2, which predominantly comprised of older GBM patients (50–70 years old), and in Asian Cohort 3. Proneural/Neural subtype was more common in Asian Cohort 2 and 3 compared to Asian Cohort 1.

Following the original four GBM-subtype classification by Verhaak^1^, several studies^12–14^ have reported refinement of the classification into three GBM-subtypes. In agreement with these studies^12–14^, we validated three robust GBM-subtypes (Classical, Mesenchymal and Proneural/Neural) but individually on six different platforms: TCGA-cohorts deposited over a decade (Affymetrix U133A = 548, Agilent Custom Array = 588 and RNA-seq = 168), and three additional Asian-cohorts (Affymetrix Human Gene 1.0 ST array = 61; Illumina HumanHT-12 v4 Expression BeadChip = 52; Agilent 4 × 44 K Whole Genome Oligo Microarray = 60) by consensus clustering^15^. To curate outlying samples, we performed silhouette width analysis^16^ to include only the core samples that were most representative of each of the three clusters (TCGA-cohorts core samples: n_1_ = 496 Affymetrix U133A, n_2_ = 523 Agilent, n_3_ = 150 RNA-seq; Supplemental 1, 2). We demonstrated that the original Verhaak 840-gene-set initially derived through a smaller number of 200 GBM samples (assayed on three gene expression platforms integrated into a single dataset)^1^, was unable to robustly cluster the larger TCGA-cohorts now available on different platforms (Fig. 1A; Supplemental 1). Through rigorous interrogation using 1500, 1000 and 500-gene-sets comprising of top ranked variable genes using maximum median absolute deviation (MAD) score, in systematic genomic simulations by consensus hierarchical clustering derived from training set (n_1_ = 496, Caucasian-predominant), we showed that a 500-gene-set was sufficient to recapitulate three GBM-subtypes (Fig. 1B, Supplemental 2) in two Caucasian-predominant validation sets (n_2_ = 523 Agilent, n_3_ = 150 RNA-Seq, TCGA cohorts) and three Asian-cohorts (core samples: Asian Cohort 1− n_4_ = 51 Affymetrix Human Gene 1.0 ST array; Asian Cohort 2 − n_5_ = 45 Illumina HumanHT-12 v4 Expression BeadChip; Asian Cohort 3 − n_6_ = 59 Agilent 4 × 44 K Whole Genome Oligo Microarray, Fig. 2D–F). Consensus hierarchical clustering uses repeated subsampling and clustering to calculate the consensus of these repetitions, which is robust relative to sampling variability, and is also the same methodology employed in TCGA-classification of tumor subtypes^17^ (Broad Institute methodology, Supplemental 2). Additionally, consensus hierarchical clustering by default performs 1000 bootstrap iterations which confirms the robustness of the clustering. The 500-gene-set was curated from the top 500 most variable genes in expression, using maximum MAD score, as a robust measure of variability to rank genes with expression values of maximal variation across the samples. Only 108 genes were common between Verhaak’s 840-gene-set and the 500-gene-set, remaining 392/500 genes were unique (Fig. 1C).

We compared the results of consensus hierarchical clustering using Verhaak’s 840-gene-set and the 500-gene-set across all six patient cohorts of GBM tumors profiled on different platforms (Fig. 2B,D–F, Supplemental 1, 2Ciii,D,E). Clusters formed using Verhaak’s 840-gene-set composed of a small, unstable 4^th^ cluster (k = 4) and less clear clusters with k = 3. In contrast, the 500 gene-classifier formed three clear clusters (Fig. 2B).

We mapped the subtype identity of each patient tumor in the TCGA training set (n_1_ = 496), comparing the GBM-subtype nomenclature defined by Verhaak’s 840-gene-set and our 500 gene-classifier (Supplemental 3A). Our analyses demonstrated that the original Proneural and Neural subgroups defined by Verhaak’s 840 gene-set, now formed a single cluster (Proneural/Neural subtype) using the 500 gene-classifier (Supplemental 3A). The remaining two clusters matched the subgroups defined by Verhaak’s GBM-subtype nomenclature of Classical subtype and Mesenchymal subtype (Supplemental 3A). Subtype-specific genes derived from each of the three GBM-subtypes defined by 500 gene-classifier, were enriched in different signaling and metabolic pathways (p value <0.05 by Fisher Exact Test, Fold-change |FC| > 2) by Ingenuity pathway analyses (Supplemental 3B, 4). As a validation, we used a gene-set comprising of 500 randomly selected genes to perform a consensus clustering analyses of the TCGA training set, the three clusters could not be created, supporting that the 500 gene-classifier was essential to construct the three GBM-subtypes (Supplemental 3C). *Seventeen percent* (32/189 from Verhaak’s cohort) of GBM tumors had original subtype nomenclature reclassified by 500 gene-classifier (Fig. 1D). Of the tumors which had subtype reclassified, majority (23/32, 71.9%) were revised to Mesenchymal subtype, from Proneural/Neural (37.5%) and Classical subtype (34.4%).

Of all three TCGA cohorts, 157 patient tumors overlapped in all cohorts (Fig. 2A), of which 119 were core samples. We compared the 500 gene-classifier on these 119 core samples across all 3 TCGA platforms (Affymetrix U133A, Agilent, RNA-seq), only 1 tumor was mismatched in subtype identity across all three platforms. For the remaining tumors (118/119), subtype identity agreed across three platforms (78.8%) and two platforms (21.2%). The highest agreement was between Agilent and RNA-seq platforms (88.2% agreement), followed by Affymetrix U133A and Agilent platforms (86.5% agreement), and Affymetrix U133A and RNA-seq platforms (80.7% agreement).

Additionally, we tested another independent 150-gene-set reported by Wang^12^ by consensus hierarchical clustering on the TCGA training set (n_1_ = 496). The clustering effect was less distinct using Wang’s 150-gene-set (Supplemental 2F) compared to the 500-gene-set (Fig. 1B). Only 21 genes were common between Wang’s 150-gene-set and our 500-gene-set (Supplemental 2F).

### Tumor subtype patterns among populations of Caucasians and Asians

**Figure 3:**
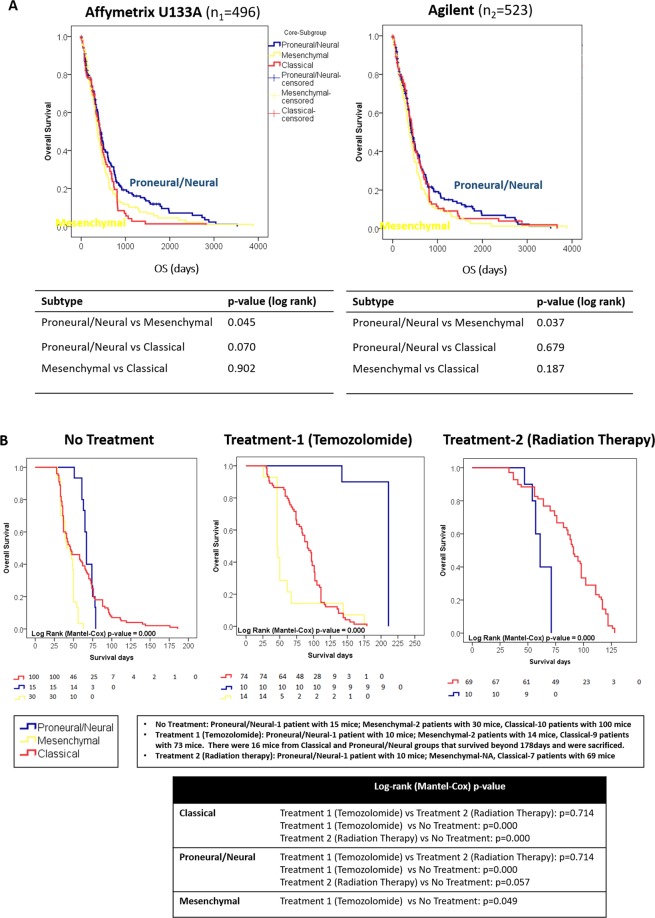
Treatment response to individual effects of temozolomide versus radiation therapy among patient-derived orthotopic mouse models of three glioblastoma subtypes. (**A**) Survival data from patients who received a *heterogenous mixture of treatment regimens* from the two largest TCGA cohorts (n_1_ = 496, n_2_ = 523) illustrated a better overall survival outcome for Proneural/Neural (previously defined by IDH-mutant and younger patient age, which were reported to have better prognosis^4^) versus Mesenchymal subtype (p < 0.05). Subtype-specific patient survival outcome was overall poor and reflected the net result of a mixture of treatment regimens with/without surgical resection. Patient survival pattern paralleled some similarity to treatment naïve patient-derived orthotopic xenograft (PDOX) mice which received no treatment, although as expected mice survival was remarkably shorter than patient survival. (**B**) As a proof-of-concept, in subtype-specific PDOX mice, Classical-subtype which was well-represented by 10 PDOX models in our cohort, demonstrated no survival difference comparing individual effects of radiation therapy versus temozolomide (p = 0.71). Though preliminary, a PDOX model of Proneural/Neural-subtype demonstrated survival benefit temozolomide (n = 10, p < 0.001) compared to untreated mice (n = 15), while no survival benefit was observed with radiation therapy (n = 10, p = 0.06) compared to untreated mice (n = 15).

Caucasians appeared to have a predominant Proneural/Neural subtype (41.2%) in the TCGA training set (Caucasian = 430 patients with assessable ethnicity data, Fig. 2C). Among the three Asian-cohorts surveyed, this subtype was less predominant in Asian Cohort 1 (Koreans, 17.6%, Fig. 2D), more common in Asian Cohort 2 (Koreans, 33.3%, Fig. 3E) and Asian Cohort 3 (Chinese, 37.3%, Fig. 2F). In contrast, Classical subtype appeared to be the more predominant subtype in Asian Cohort 1, contributing up to 49% (Koreans, Fig. 2D), less common in Asian Cohort 2 (Koreans, 28.9%, Fig. 2E) and Asian Cohort 3 (Chinese, 23.7%, Fig. 2F). This subtype was less common among Caucasians in the TCGA training set (21.2%, Fig. 2D). Mesenchymal subtype appeared more consistent in proportion among all cohorts surveyed (TCGA training set, 37.7% and Asian Cohorts 1–3 ranging 33.3%, 37.8%, 39% respectively, Fig. 2). It is important to note that the demographics of each of the Asian-cohorts vary and we did not have the large-scale resource to interpret the findings in conjunction with multi-dimensional genomics data. However, these larger Asian-cohorts were possibly a more accurate representation of the subtype distribution among Asian GBMs compared to the smaller Asian TCGA cohort (n = 13, Supplemental 3D). It should be highlighted that the sample size for the Caucasian-predominant TCGA-cohort was very much larger. This was a descriptive observation, and no conclusive findings can be made as other correlates such as mutational status were not analyzed, populations were demographically distinct and the Asian-cohorts were much smaller than TCGA-cohorts.

### Treatment response in preclinical models of each GBM subtype

Most GBM prediction models reported^18–22^ were prognostic-based. However, almost all GBM patients die, with the best 2-year overall survival of about 25%^9–11^, which limited the clinical utility of prognostic-prediction models. Further, in these prognostic models, patients had received a *heterogenous mixture of treatment regimens*, which posed additional challenges for interpretation and translation into clinical application^19,21,22^. We postulated that survival differed by subtypes in response to individual effects of each single-agent therapy, for the two treatment modalities used in the standard of care for GBM – temozolomide and radiation therapy. In the clinical context, patients had mostly received a heterogenous mixture or combination of treatment regimens with or without surgical resection of varying extent. Using large retrospective clinical sets from TCGA with assessable outcome data for good statistical power, we stratified these patients into three GBM-subtypes using the 500 gene-classifier. Subtype-specific patient survival outcome was similarly poor and reflected the net result of a mixture of treatment regimens with or without surgical resection of varying extent (Fig. 3A). Next, we questioned if PDOX mice bearing tumor xenografts of each of the three GBM-subtypes, differed in treatment response to individual arms of radiation therapy versus temozolomide, in context of GBM-relevant therapies. This allows the treatment response of a single tumor subtype from each patient, to be studied in three different settings of no treatment, temozolomide monotherapy and radiation monotherapy, to investigate if there exist temozolomide-responsive or radiotherapy-responsive subtypes, without surgical resection (lessening tumor burden) of these tumors in mice or a mixture of therapies confounding the interpretation of survival data.

As a proof-of-concept, we showed in a large cohort of 321 PDOX GBM mouse models derived from 13 treatment-naïve patient GBM tumors from Asian Cohort 1 (Fig. 3B), that Classical subtype demonstrated no survival difference comparing radiation therapy with temozolomide treatment (p = 0.71). Classical subtype PDOX models displayed similar survival advantage with radiation therapy (p < 0.001) or temozolomide (p < 0.001) compared with untreated mice (Fig. 3B). We have a larger representation of Classical subtype PDOX models (No Treatment: 10 patient models, Treatment 1-Temozolomide: 9 patient models, Treatment 2-Radiation Therapy: 7 patient models), compared to the other two subtypes, which may be partly contributed by the larger proportion of Classical subtype tumors in Asian-cohort 1 (Fig. 2D) compared to Caucasian-cohorts (Fig. 2A). Though preliminary, among PDOX mice implanted with Proneural/Neural GBM (PDOX Model 7, Supplemental 5) from one patient tumor model, we observed significant survival difference between temozolomide-treated and radiation-therapy-treated mice (Fig. 3B). Treated mice from PDOX Model 7 demonstrated significant survival benefit with temozolomide (n = 10, p < 0.001) compared to untreated mice (n = 15), while no survival benefit was observed with radiation therapy (n = 10, p = 0.06) compared to untreated mice (n = 15, Fig. 3B). In two Mesenchymal subtype PDOX models (PDOX Model 9 and 10), temozolomide treatment (n = 14) did not confer significant survival benefit in mice compared to untreated mice (p = 0.05, n = 30, Fig. 3B).

It was unclear if Classical subtype GBM tumors have a selective growth advantage and tumor take-rate in PDOX models compared to other subtypes, and we were limited by the smaller number of Proneural/Neural and Mesenchymal subtype PDOX models. But among these few PDOX models, though preliminary, temozolomide appeared to confer a significant and selective survival advantage to the PDOX model of Proneural/Neural subtype compared to radiation therapy (p < 0.001, Fig. 3B), while for two PDOX models of Mesenchymal subtype, treatment with temozolomide did not give any significant benefit compared to untreated mice (p = 0.05, Fig. 3B). Treatment-naïve mice of each subtype also exhibited different survival characteristics (p < 0.001). The number of models in each subtype was dependent on the patients who presented consecutively during the period of PDOX creation, available patient tissue samples for injection and post-treatment mouse survival data pooled from various studies^23,24^ involving this panel of PDOX models from Asian Cohort 1, and therefore not equally distributed among subtypes. We acknowledge this limitation, but our goal in this study was to capture the most comprehensive cohort of mice with patient tumors injected from the Asian validation cohort. Each of the 13 patient tumors had an average of 11, 8 and 10 mice (replicates) per model respectively in No Treatment, Treatment 1 (Temozolomide) and Treatment 2 (Radiation Therapy) groups (Supplemental 5), and the survival curves (Fig. 3A) reflected a collection of all the mice within each subtype. This study was not designed with a fixed number of mice from all three subtypes to undergo different therapies, therefore for the group of mice that received radiation therapy, there were only two subtypes available. As the data was extracted from post-treatment mouse survival data pooled from various studies^23,24^ involving this panel of PDOX from Asian Cohort 1, there were no animals treated with combined temozolomide-radiation therapy in these studies, hence this data was not available to be included in our current survival analyses. However, the observed differences of the treatment response in mice, highlighted a gap to be addressed in larger scale studies to investigate the subtype-specific treatment effects for appropriate patient selection in subtype-based therapy.

Recent emerging evidence supports the importance of patient selection based on subtypes in clinical trials and therapeutic drug development^25^. Survival data from patients who received a *heterogenous mixture of treatment regimens* (chemotherapy or radiation therapy or combination of chemotherapy with radiation therapy, with or without surgical resection), from the two largest TCGA cohorts (Affymetrix n_1_ = 496, Agilent n_2_ = 523, Fig. 3A) illustrated a better overall survival outcome for Proneural/Neural (previously defined by IDH-mutant and younger patient age, which were reported to have better prognosis^4^) versus Mesenchymal GBM (p < 0.05). The patient survival pattern paralleled some similarity to treatment naïve PDOX mice which received no treatment, although as expected mice survival was remarkably shorter than patient survival. Most survival data from GBM patients^19,21,22^, including our Asian cohorts, reflected the outcome of a *heterogenous mixture of treatment regimens* and different extent of surgical resection of tumor bulk, which posed further challenges to meaningfully interpret patient data for subtype-specific responses to individual effects of temozolomide versus radiation therapy, in context of GBM-relevant therapy. Therefore, we sought post-treatment mouse survival data to provide some biological evidence for new clinical insights, investigating the individual effects of each single-agent therapy, for the two common treatment modalities in the standard of care in GBM.

## Discussion

Here, we present clinically relevant biological data in a proof-of-concept study investigating TCGA-cohorts to derive a gene-classifier that can be applied across different gene profiling platforms and population groups. Our findings from this study, if done on a larger scale, may be useful in clinical outcome prediction and patient selection for trials based on subtyping. We validated three GBM-subtypes using our gene-classifier on six different platforms among Caucasian, Korean and Chinese populations. This will provide institutions profiling patient tumors on various platforms internationally, with a model to predict GBM-subtypes in adults. We did not find this gene-classifier useful in subtyping pediatric GBMs (Supplemental 3F), which previously^26^ have been reported to be a different disease from adults.

The rapidly emerging field of epigenomics and the explosion of large-scale genomics have dissected GBM tumors into complex molecular subgroups^7^ and provided insights on GBMs across age groups^26^. This study neither attempted nor was designed to reconcile all levels of genomic data available for GBM^27–30^, but rather to harmonize and derive a robust subtype-prediction model, which can be relevant to Caucasian, Korean and Chinese patient populations of adult GBM, independent of gene profiling platforms. To address the ground truth of the subtype of each patient tumor correlating with multi-dimensional genomic data such as MGMT methylation status^9,31^ and IDH1/2 mutations^4^, and more recently, TERT and ATRX mutations^32^, will require larger resources and scale of study. We acknowledge this limitation. Future studies incorporating these elements into the 500 gene-classifier to apply to the issues of ethnicity-based analyses will be useful to enrich the current classification scheme. However, our finding of three transcriptomic GBM subtypes is consistent with other post-Verhaak’s studies that reported three GBM subtypes at the proteomic level^13^, and another study that described three GBM subtypes using integrated data from gene expression clustering with Verhaak’s 840 gene and methylation data^14^. The study reporting integrated subtypes^14^ found four dispersed transcriptomic clusters using Verhaak’s 840 gene-set, which is consistent with our findings with Verhaak’s gene set. The relevance of molecular cancer signatures across ethnic groups remains an under-investigated field - whether cancer signatures derived from Caucasian patients can be applied on other large ethnic groups such as Asians, contributing to a majority 60% of the world’s ~7 billion population. While high throughput genomic data rapidly accumulates, the gap widens between genomic science and clinical translation of genomic information to patient care.

Nonetheless, it is difficult to meaningfully interpret Asian GBM-subtype distribution based on the small sample size of Asians in TCGA training set (n = 13). We therefore sought additional larger Asian-cohorts (core samples: n_4_ = 51, n_5_ = 45, n_6_ = 59) to validate our gene-classifier. Limitations include variation of demographics among the Asian-cohorts vary and lack of large-scale multi-dimensional genomics resource. Here, we present a descriptive observation, since other correlates such as mutational status were not analyzed, populations were demographically distinct and the Asian-cohorts were much smaller than TCGA-cohorts. Among the cohorts surveyed, Caucasian-predominant TCGA training set was Proneural/Neural subtype predominant (41.2%). This subtype was less common in Asian Cohort 1 (Koreans, 17.6%), more common in Asian Cohort 2 (Koreans, 33.3%) and Asian Cohort 3 (Chinese, 37.3%). Classical subtype appeared to be the more predominant subtype in Asian Cohort 1 (Koreans, 49%), less common in Asian Cohort 2 (Koreans, 28.9%) and Asian Cohort 3 (Chinese, 23.7%). This subtype was less common among Caucasians in the TCGA training set (21.2%). Mesenchymal subtype appeared more consistent in proportion among all cohorts surveyed. We found another cohort of 88 Chinese GBM patients (RNA-seq), comprising of Mesenchymal subtype (41%), Classical subtype (36%), and Proneural/Neural subtype (23%)^33,34^.

To our knowledge, while there are studies comparing glioma types of different WHO grading among ethnic populations (lower survival among non-Hispanic whites for GBM)^35^, there are no studies investigating GBM-subtype distribution among ethnic groups. Incidence of GBM is higher in the male gender and among ethnic groups of White and non-Hispanics^36^. The incidence rate of GBM among Blacks, Asians/Pacific Islanders, and American Indians/Alaskan Natives was substantially lower compared to non-Hispanic whites^37^. Our findings represent an early effort to survey and describe GBM-subtype distribution between patient populations of two ethnic groups of Caucasians and Asians (2 Korean-cohorts and 1 Chinese-cohort). We report a TCGA-derived subtype-prediction model which can be useful for institutions profiling patient tumors on various platforms among populations of Caucasian, Korean and Chinese patients to predict GBM-subtypes. This approach can be applied to other tumor types with big data deposited in TCGA, investigating TCGA-cohorts to understand correlation with Asian ethnicity. Larger scale studies might be useful in clinical outcome prediction and patient selection for trials based on subtyping for other major cancers. Our gene-classifier is validated among Caucasian and Asian populations and can be relevant in understanding the subtype patterns among ethnic populations if applied on a larger scale. Subtype patterns may be relevant for translating subtype-specific trials across international patient populations.

In the clinical context, patients had mostly received a heterogenous mixture or combination treatment regimens with or without surgical resection of varying extent. Using large retrospective clinical sets from TCGA with assessable outcome data for good statistical power, subtype-specific patient survival outcome was similarly poor and reflected the net result of a mixture of treatment regimens with or without surgical resection of varying extent. As a proof-of-concept, in subtype-specific PDOX mice, Classical subtype demonstrated no survival difference comparing the individual effects of radiation therapy versus temozolomide monotherapies. We also observed that temozolomide did not confer significant survival benefit in two Mesenchymal subtype PDOX models, compared to untreated mice. Though preliminary, a PDOX model of Proneural/Neural subtype demonstrated significantly improved survival with temozolomide compared to radiation therapy. Our approach is clinically relevant. It compares the individual effects of temozolomide versus radiation therapy, the two most common treatment modalities in GBM, among PDOX models without surgical resection (lessening tumor burden) of these tumors in mice or a mixture of therapies confounding the interpretation of survival data. It allows the treatment response of a single tumor subtype from each patient, to be studied in three different settings of no treatment, temozolomide monotherapy and radiation monotherapy, to investigate if there exist temozolomide-responsive or radiotherapy-responsive subtypes. Our study was limited in design as the data was extracted from post-treatment mouse survival data pooled from various studies^23,24^ involving this panel of PDOX from Asian Cohort 1, and fewer PDOX models on Proneural/Nerual and Mesenchymal subtypes. However, our Classical PDOX models were well-represented, and our sample size of 13 PDOX models from Asian Cohort 1 was appropriately-sized for the funding and manpower resources allocated for each of these PDOX projects in the PDOX modelling field. Creation of PDOX models is a lengthy and costly process. PDOX models are still the preferred cancer model over culture-based spheroids or slice cultures. In 2016, the US National Cancer Institute (NCI) has decided to stop screening most drugs using the NCI-60, its panel of 60 human cancer cell lines grown in culture, after more than 25 years of heavy use by worldwide researchers, and is developing PDOX models that better mimic the human counterpart. Specifically, a targeted approach of testing drugs with subtype-specific models, which we illustrate here, but with extended collaborations to various international groups studying GBMs, and each research group with a defined focus on different subtype-specific models, may be a cost- and time-effective approach to team science using PDOX models, without compromising the value of data we can derive from PDOX models. Our observations highlight a gap to be addressed in larger scale PDOX studies to prospectively investigate the differential treatment response for subtype-based GBM patient selection.

## Methods

### Patients and tumor samples

GBMs and normal brain tissue samples were obtained from The Cancer Genome Atlas (TCGA) Research Network with data available on three different platforms RNA-seq, Affymetrix HT-HG-U133A arrays and custom designed Agilent arrays. We downloaded open access TCGA Level-3 data on 4 Feb 2015 using the TCGA data portal. Level-3 data has been already normalized for downstream analyses. We have chosen genes that were common on all 3 platforms for the analyses. We used 548 GBMs and 10 normal samples (Affymetrix HT-HGU133A array) and 588 GBMs and 10 normal samples (Agilent array) and 168 GBMs and 5 normal samples (RNA-seq) to identify differentially expressed genes (DEGs) between GBMs and normal brain tissue samples. Figure 2 and Supplemental 3D summarized the patient demographic information on age of diagnosis and ethnicity with the molecular subtype. We used 548 GBMs from Affymetrix HT-HGU133A as a training set for constructing consensus average linkage hierarchical clustering and we used other two platform datasets (588 tumors on Agilent and 168 tumors on RNA-seq) as validation sets. A total of 595 non-overlapping tumor samples were found in all 3 GBM cohorts in TCGA (Affymetrix HT-HGU133A, Agilent and RNA-seq). Asian Cohort 1 of 61 adult GBM patients was derived by combining a previously published set of 58 patients (GSE42670)^23^ with 3 additional new patients from the same single institution. Asian Cohort 2 comprised of 52 adult GBM patients from Yonsei University Hospital, normalized gene expression values were provided for Verhaak’s^1^ 840 genes and 500 genes in our gene-classifier (Gene List A). Asian Cohort 3 comprised of 60 adult GBM patients from a previously published data set (GSE74187) Tian Tan Hospital, China^38^. Gene expression profiling experiments for Asian Cohort 1 were conducted using Affymetrix Human Gene 1.0 ST array according to manufacturer’s protocol^23^. Gene expression profiling experiments for Asian Cohort 2 were conducted using Illumina HumanHT-12 v4 Expression BeadChip according to manufacturer’s protocol. Gene expression profiling experiments for Asian Cohort 3 were conducted using Agilent 4 × 44 K Whole Genome Oligo Microarray according to manufacturer’s protocol^38^. Pediatric cohort of 25 patients is obtained from GSE19578^26^, the gene expression profiling experiments were performed on Affymetrix Human Genome U133 Plus 2.0 Array (HG-U133 Plus 2). TCGA training set comprised of 475 patients with identifiable ethnicity (core samples), of which 430 were Caucasians, 13 Asians, and 32 African-Americans (Supplemental 3D). This study comprised of analyses of retrospective data-sets deposited in the public domain, and ethics approval was obtained from Centralized Institutional Review Board at SingHealth. The other relevant ethics approval for each of these retrospective data sets can be found in the respective studies cited. Asian Cohort 2 study was approved by the institutional review board of Severance Hospital, Yonsei University College of Medicine and conformed to the requirements of the Declaration of Helsinki. These patients provided written informed consent.

### Data filtering for identification of DEGs

Several filters were applied to identify most tumour specific relevant genes for clustering. We identified differentially expressed genes between GBMs and normal brain tissue samples in all three datasets separately using ANOVA comparisons. A cut-off of P < 0.05, Q < 0.05 and |FC| > 2 were used which identified 2723 differentially expressed genes in Affymetrix HT-HGU133A dataset and 3284 differentially expressed genes in Agilent array and 6753 differentially expressed genes in RNA-seq dataset. We overlapped three sets of differentially expressed genes and identified a final set of 1500 genes (Gene List B). The second filter was applied using median absolute deviation score (MAD score) higher than 0.5, which eliminated genes with low variability across patients. Three different simulations were performed using differentially expressed genes that were ranked based on the MAD score. Top 1500, 1000 and 500 genes were selected based on the high MAD score and was used for consensus hierarchical clustering (Supplemental 2). The clustering results were compared based on these three gene sets. The minimal gene set of 500 genes was sufficient for identifying GBM subtypes.

### Consensus hierarchical clustering

Consensus hierarchical clustering is a resampling-based clustering algorithm that provides a way to represent the clustering consensus across multiple runs of a clustering algorithm and helps to assess the stability of the discovered clusters. We used hierarchical clustering with agglomerative average linkage, as our basis for consensus clustering, to detect robust clusters^15^. The distance metric was 1-(Pearson’s correlation coefficient) and the procedure was run over 1000 iterations and a subsampling ratio of 0.8 was used to cluster the training cohort of 548 GBM samples with 500 reliably-expressed gene set identified based on various filtering.

### Identification of gene expression-based subtypes

We used consensus average linkage hierarchical clustering^15^ approach to identify subgroups based on the 500 reliably-expressed genes identified using the above filter. We used Pearson’s correlation as the distance metric and 0.8 as subsampling ratio using the training set of 548 GBM’s. Over 1000 iterations were performed to identify the stable clusters using the top 500 genes. Silhouette width analysis using (R-package: Silhouette) was performed to identify the core samples that were most representative of the clusters^16^. ANOVA comparisons between all three subgroups both pairwise and in combination were performed to identify subtype specific genes which were highly expressed in specific subtype.

### Statistical analyses

Statistical Analysis was performed using Partek Genomics Suite software, version 6.6 Copyright ©; 2016 Partek Inc., St. Louis, MO, USA. Kaplan-Meier Survival Analysis was performed using PASW Statistics 18, Release 18.0.0 (July 30, 2009).

### Identification of subtype specific pathways

We used Ingenuity Pathway Analysis (IPA) to identify significant pathways (Supplemental 4, 3B) associated with the subtype specific genes (Gene List C). The top ten canonical pathways were generated in IPA and the significant pathways (p < 0.05; calculated by Fisher’s exact test) were chosen for each subgroup to identify signaling pathways that are specific to each subgroups.

### Orthotopic xenograft animal model

Animal experiments were approved by the Institutional Review Board and conducted in accordance with the “National Institutes of Health Guide for the Care and Use of Laboratory Animals” (NIH publication). Tumors were classified as GBM based on WHO criteria after review by pathologists. Parts of the surgical samples were enzymatically dissociated into single cells, following the procedures previously reported^23^. Dissociated GBM cells were cultured in neurobasal media with N2 and B27 supplements (0.5X each; Invitrogen) and human recombinant bFGF and EGF (25 ng/ml each; R&D Systems) (NBE condition). Acutely dissociated GBM cells were stereotactically (2 mm left and 1 mm anterior to the bregma, 2 mm deep from the dura) injected into the brains of immune-compromised NOD/SCID Il2rg^−/−^ (NOG) mice within 12 hours after surgery (2.5 × 10^4^–1.0 × 10^5^ cells in 10 ml HBSS for each mice, n = 4–19 for each sample). Mice with the reduction of the total body weight (>20%) were sacrificed, and brains were processed for paraffin or frozen section.

### Whole-brain *In Vivo* irradiation and temozolomide chemotherapy

Orthotopic xenograft tumors were made as described above, using primarily cultured GBM cells that had short term *in vitro* culture in the NBE condition (*in vitro* passage <6, 2.0 × 10^5^ cells for each animal). Treatments were started at the half of the median survival length of the orthotopic xenograft animal models. The reduction of the total body weight (>20%) was regarded as mortality. Whole brain 2 Gy X-irradiations were applied daily for 5 days (total 10 Gy) using a blood irradiator (IBL-437C, CIS-US). Mouse bodies were shielded with a custom-made lead shield device. Temozolomide (65 mg/kg) was orally administrated daily.

## Supplementary information

Supplementary Figures and Tables

4A

4B

4C

Gene List A_500 Gene Classifier

Gene List B_1500 Gene-Set

Gene List C_Subtype Specific Genes

## Data Availability

The Cancer Genome Atlas (TCGA) Research Network; Accession Codes- GSE42670, GSE19578, GSE74187.
