# Supplementary material for: Relevance of a TCGA-derived Glioblastoma Subtype Gene-Classifier among Patient Populations
Source: Sci Rep. 2019 May 15;9:7442. doi: 10.1038/s41598-019-43173-y (PMC6520485; doi:10.1038/s41598-019-43173-y)
Supplement: Supplementary file 1 — Supplementary Figures and Tables [file 41598_2019_43173_MOESM1_ESM.pdf]

Relevance of a TCGA-derived Glioblastoma Subtype Gene-Classifier among Patient Populations

Authors: Wan-Yee Teo[1-8*] (MD, PhD. *Corresponding Author*), Karthik Sekar[1,3] (MSc), Pratap Seshachalam[1,3] (MSc), Jianhe Shen[5,6] (BSc), Wing-Yuk Chow[5,6] (PhD), Ching C Lau[5-7] (MD, PhD), HeeKyoung Yang[9] (PhD), Junseong Park[10] (PhD), Seok-Gu Kang[10] (MD, PhD), Xiaonan Li[5-7,11,12] (MD, PhD), Do-Hyun Nam[9] (MD, PhD), Kam M Hui[1,3-4,8,13] (PhD)

ONLINE ONLY SUPPLEMENT
Supplementary Figures
Supplementary Tables

Legends for Supplementary Figures & Supplementary Tables

<u>Supplemental 1</u>

A. Identification and validation of previously identified four GBM subtypes identified with the bigger cohort of 548 Affymetrix U133A array TCGA samples using previously identified predictive 840 genes set.

B. Consensus Clustering CDF for k=2 to 10 are shown.

<u>Supplemental 2</u>

A. Bioinformatics pipeline to identify differentially expressed genes between GBM and normal samples from all three cohorts of TCGA data.

B. Venn diagram showing 1500 overlapping differentially expressed genes between GBM and normal samples from all three cohorts of TCGA data.

C. Affymetrix U133A - TCGA

(i) Using 1500 genes: Identification of three GBM subtypes based on unsupervised consensus hierarchical clustering using 548 TCGA Affymetrix U133A samples (Training set) with **1500 genes** differentially expressed gene set. The clustering was shown ranging from k=2 to k=10. Silhouette plot for identification of core samples was shown for most stable k=3 cluster.

(ii) Using 1000 genes: Identification of three GBM subtypes based on unsupervised consensus hierarchical clustering using 548 TCGA Affymetrix U133A samples with top **1000 genes** differentially expressed gene set chosen based on highest MAD score. The clustering was shown ranging from k=2 to k=10. Silhouette plot for identification of core samples was shown for most stable k=3 cluster.

(iii) Using 500 genes:

a. Identification of three GBM subtypes based on unsupervised consensus hierarchical clustering using 548 TCGA Affymetrix U133A samples with top **500** differentially expressed gene set chosen based on highest MAD score. The clustering was shown ranging from k=2 to k=10. Silhouette plot for identification of core samples was shown for most stable k=3 cluster.

b. Identification of three GBM subtypes using 498 core set of TCGA Affymetrix U133A samples with predictive **500** gene set. The clustering was shown ranging from k=2 to k=9. Silhouette plot for identification of core samples was shown for most stable k=3 cluster.

D. Agilent Array - TCGA

a. Validation of predictive 500 genes set and Identification of three GBM subtypes based on unsupervised consensus hierarchical clustering using 588 TCGA Agilent array samples as a validation data set. The clustering was shown ranging from k=2 to k=10. Silhouette plot for identification of core samples was shown for most stable k=3 cluster.

b. Identification of three GBM subtypes using 523 core samples with predictive 500 gene set. The clustering was shown ranging from k=2 to k=9. Silhouette plot for identification of core samples was shown for most stable k=3 cluster.

E. RNA-Seq – TCGA

a. Validation of predictive 500 genes set and Identification of three GBM subtypes based on Unsupervised Consensus hierarchical clustering using 168 TCGA RNA-Seq data as a validation set. The clustering was shown ranging from k=2 to k=10. Silhouette plot for identification of core samples was shown for most stable k=3 cluster.

b. Identification of three GBM subtypes using 150 core samples with predictive 500 genes set. The clustering was shown ranging from k=2 to k=9. Silhouette plot for identification of core samples was shown for most stable k=3 cluster.

F. Using Wang, *et al.*[12] 150 Gene Model

<u>**Supplemental 3**</u>

A. Comparing 4 GBM subtypes that were previously identified using 840 gene set and the current three subtypes identified using the 500 gene set, our analyses demonstrate that original Proneural and Neural subgroups are now classified as the same cluster using the 500 gene set.

B. Ingenuity pathway analysis (IPA) software was used to identify significant signaling and metabolic pathways. We have performed ANOVA using the three subtypes and have identified subtype-specific genes for each subtype with the cut-off of p-value < 0.05 (Fisher Exact Test) and Fold-change|FC| > 2. We have identified 195 genes specific to Subtype 1 (Mesenchymal), and 156 genes specific to Subtype 2 (Proneural/Neural) and 49 genes specific to Subtype 3 (Classical). We have performed Ingenuity pathway analyses using these subtype specific genes, 195 genes (Subtype 1-Mesenchymal), 156 genes (Subtype 2- Proneural/Neural) and 49 genes (Subtype 3- Classical) to identify top canonical pathways associated with each GBM-subtype. Mesenchymal subtype is enriched in cellular immune response, while Classical subtype and Proneural/Neural subtypes were enriched in neurotransmitter and other nervous system signalling, and intracellular and second messenger signalling as shown below. The significant pathways of three subgroups were shown in Supplemental 4.

C. Validation using randomly selected 500 genes to perform a consensus cluster analyses of TCGA training set, three subtypes could not be created, supporting the 500-gene-classifier was essential to construct the three GBM-subtypes.

D. Distribution of GBM subtypes among different age groups and ethnic groups in TCGA training set (Affymetrix),

E. Relevance of 500 Signature Gene Set Classifier among Asian Adult GBMs. Heatmap showing three GBM subtypes among 51 core samples in a cohort of 61 Asian patients (58 patients from GSE42670 and 3 new patients from the same institution). The 500 gene classifier is able to recapitulate 3 GBM subtypes in a cohort of Asian patients with GBM. Consensus clustering matrix for k=3 of 51 core samples in Asian adult GBM cohort. Silhouette plot identified 51 core samples. Consensus clustering CDF for k=2 to k=10.

F. 25 Caucasian pediatric GBMs. The 500 gene classifier was able to recapitulate three GBM subtypes in Asian adult patient GBM cohort but not pediatric GBMs in a Caucasian-predominant childhood cohort.

G. Comparison of subtype patterns across all patient populations of Caucasians, Koreans and Chinese did not reveal a consistent statistical trend, likely due to the larger TCGA Caucasian cohorts compared to smaller Asian cohorts and demographics differences.

H. Schematic

Supplemental 4

Identification of subtype specific pathways using IPA: List of differentially expressed genes between three GBM subtypes identified using ANOVA with P-value < 0.05 and |FC| > 2 shown in Gene List 4 were input into IPA to derive significant pathways (Supplementary Table A-C).

Supplemental 5

Average number of mice per patient-derived orthotopic (PDX) model for GBM derived from patients in Asian Cohort 1, in No Treatment, Treatment 1 (Temozolomide) and Treatment 2 (Radiation Therapy) groups of mice.

Supplemental Data

Supplemental 1

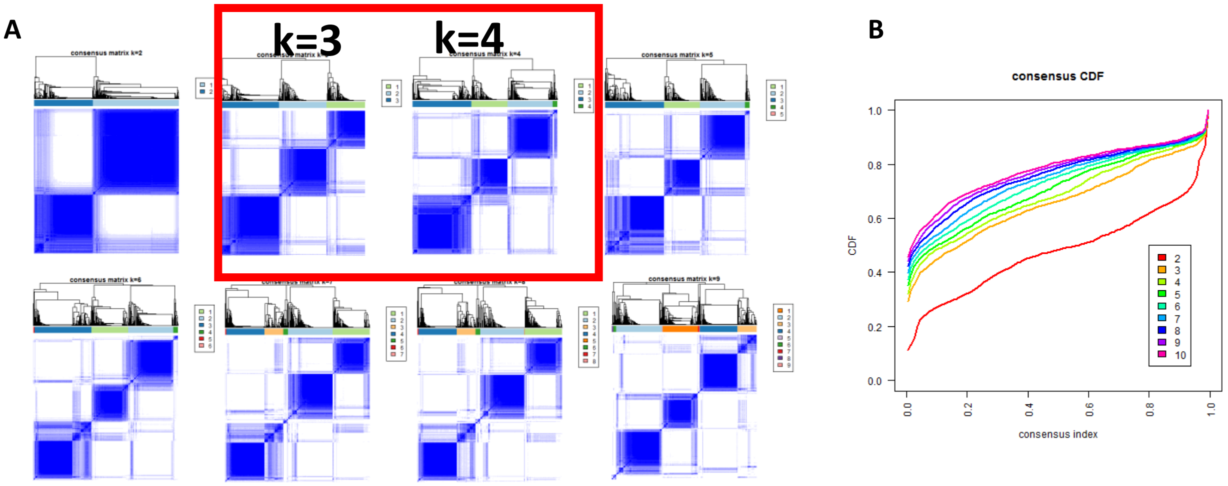

(A) Identification and validation of previously identified 4 GBM subtypes identified with the bigger cohort of 548 Affymetrix U133A array TCGA samples using previously identified predictive 840 genes set.

(B) Consensus Clustering CDF for k=2 to 10 are shown.

Supplemental 2

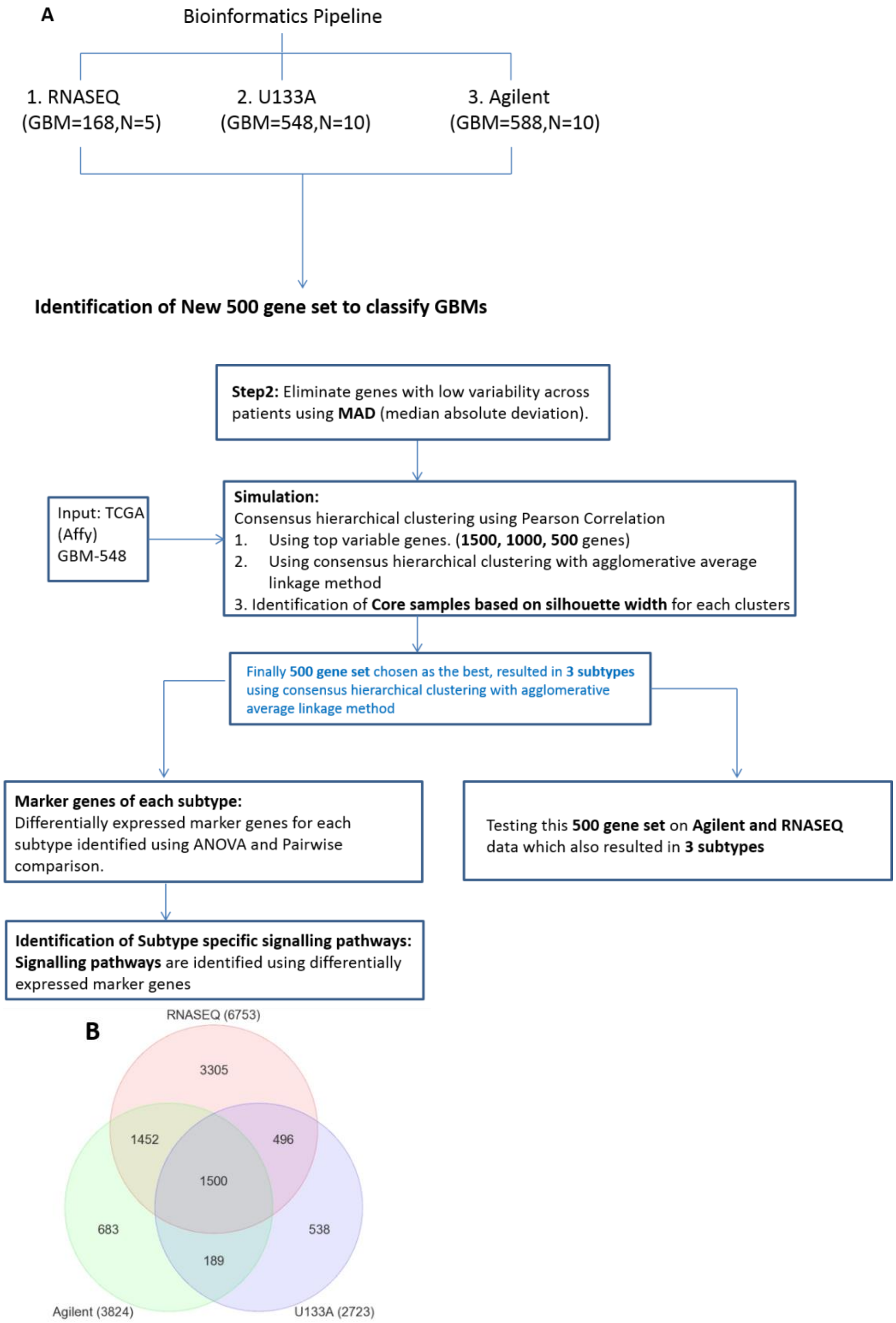

A. Bioinformatics pipeline to identify differentially expressed genes between GBM and normal samples from all three cohorts of TCGA data.

B. Venn diagram showing 1500 overlapping differentially expressed genes between GBM and normal samples from all three cohorts of TCGA data.

C. Affymetrix U133A - TCGA

(i) Using 1500 genes: Identification of three GBM subtypes based on unsupervised consensus hierarchical clustering using 548 TCGA Affymetrix U133A samples (Training set) with **1500 genes** differentially expressed gene set. The clustering was shown ranging from k=2 to k=10. Silhouette plot for identification of core samples shown for most stable k=3 cluster.

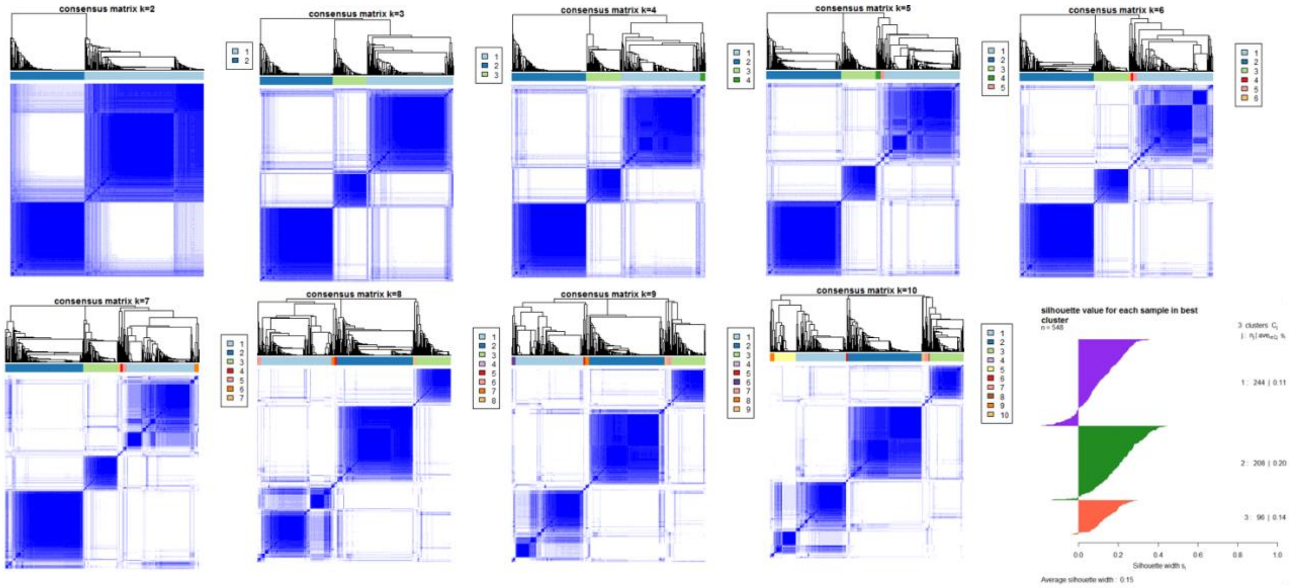

Dataset: TCGA Affymetrix-U133A data
Sample Size: 548
Genes used: 1500
Subtype identified: 3
Algorithm: Consensus hierarchical clustering using Pearson Correlation
Linkage method: Average

(ii) Using 1000 genes: Identification of three GBM subtypes based on unsupervised consensus hierarchical clustering using 548 TCGA Affymetrix U133A samples with top **1000 genes** differentially expressed gene set chosen based on highest MAD score. The clustering shown ranging from k=2 to k=10. Silhouette plot for identification of core samples shown for most stable k=3 cluster.

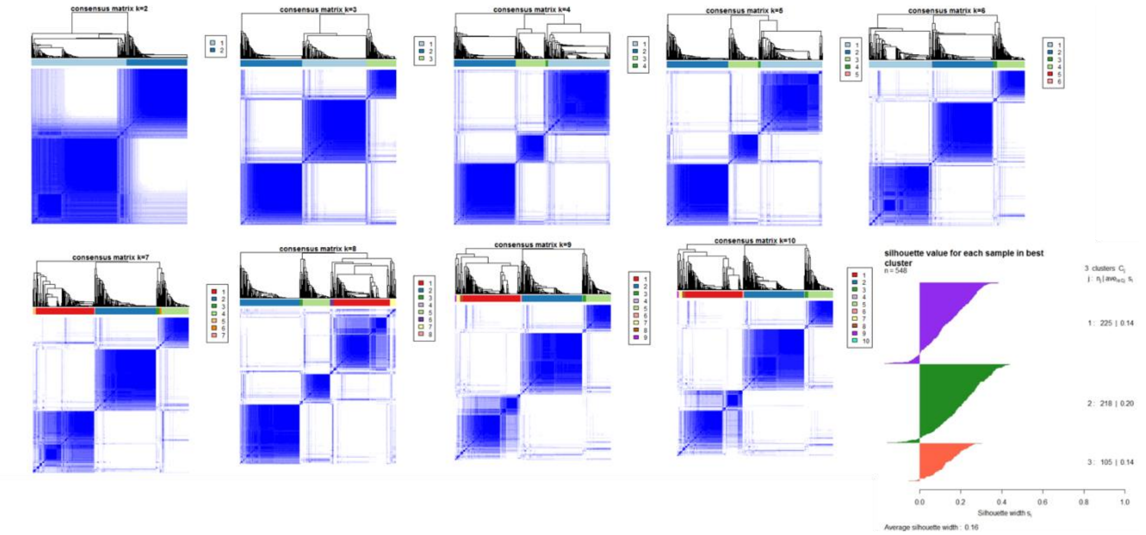

Dataset: TCGA Affymetrix-U133A data
Sample Size: 548
Genes used: 1000
Subtype identified: 3
Algorithm: Consensus hierarchical clustering using Pearson Correlation
Linkage method: Average

(iii) Using 500 genes

(a) Identification of three GBM subtypes based on unsupervised consensus hierarchical clustering using 548 TCGA Affymetrix U133A samples with top **500 differentially expressed gene set** chosen based on highest MAD score. The clustering shown ranging from k=2 to k=10. Silhouette plot for identification of core samples shown for most stable k=3 cluster.

(b) Identification of three GBM subtypes using 498 core set of TCGA Affymetrix U133A samples with predictive **500 gene set**. The clustering shown ranging from k=2 to k=9. Silhouette plot for identification of core samples shown for most stable k=3 cluster.

a

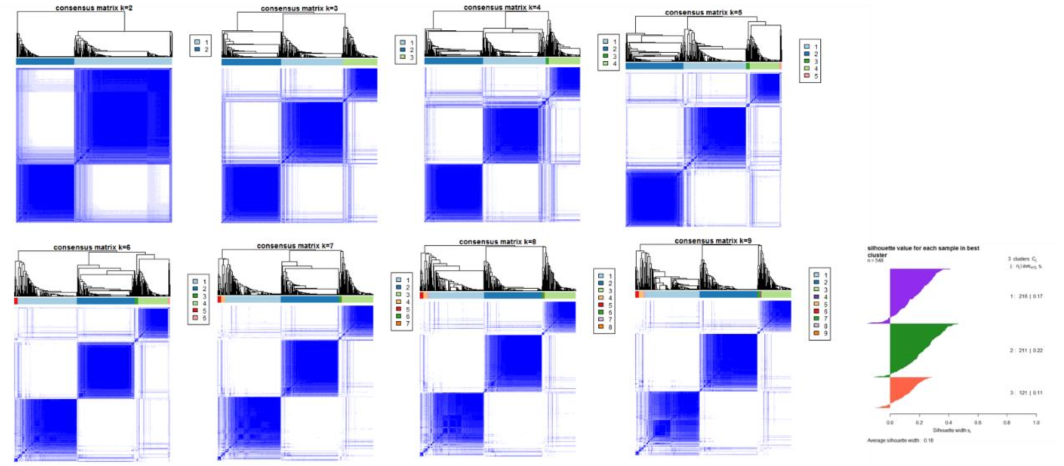

Dataset: TCGA Affymetrix-U133A data
Sample Size: 548
Genes used: 500
Subtype identified: 3
Algorithm: Consensus hierarchical clustering using Pearson Correlation
Linkage method: Average

b

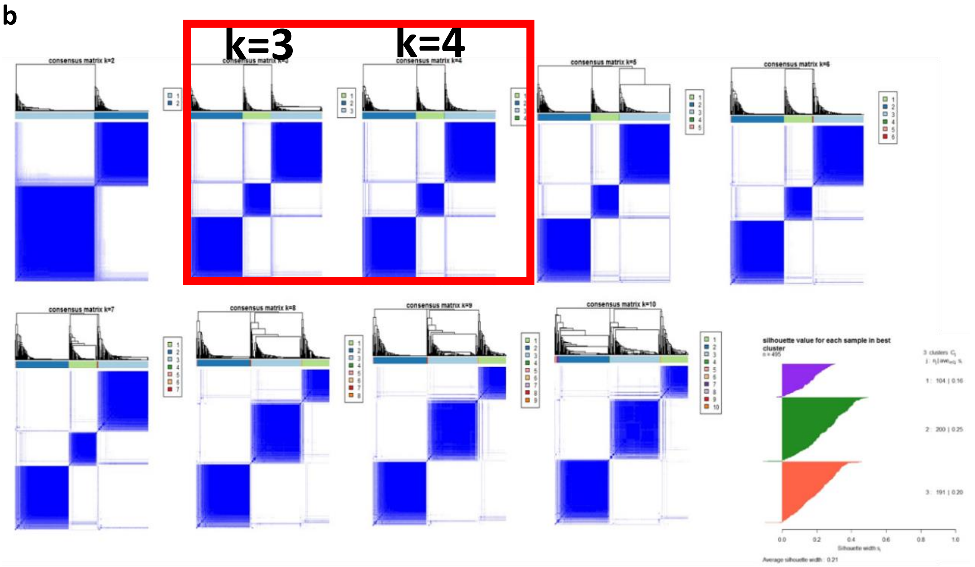

Dataset: TCGA Affymetrix-U133A data
Sample Size: 495 Core samples
Genes used: 500
Subtype identified: 3
Algorithm: Consensus hierarchical clustering using Pearson Correlation
Linkage method: Average

D. Agilent Array - TCGA

(a) Validation of predictive 500 genes set and Identification of three GBM subtypes based on unsupervised consensus hierarchical clustering using 588 TCGA Agilent array samples as a validation data set. The clustering shown ranging from k=2 to k=10. Silhouette plot for identification of core samples shown for most stable k=3 cluster.

(b) Identification of three GBM subtypes using 523 core samples with predictive 500 gene set. The clustering was shown ranging from k=2 to k=9. Silhouette plot for identification of core samples shown for most stable k=3 cluster.

a

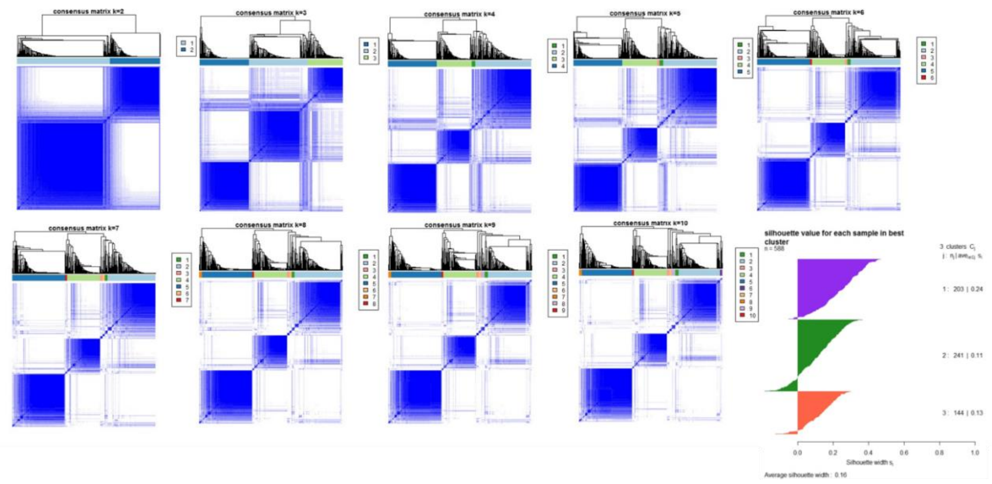

Dataset: TCGA Agilent data
Sample Size: 588
Genes used: 500
Subtype identified: 3
Algorithm: Consensus hierarchical clustering using Pearson Correlation
Linkage method: Average

b

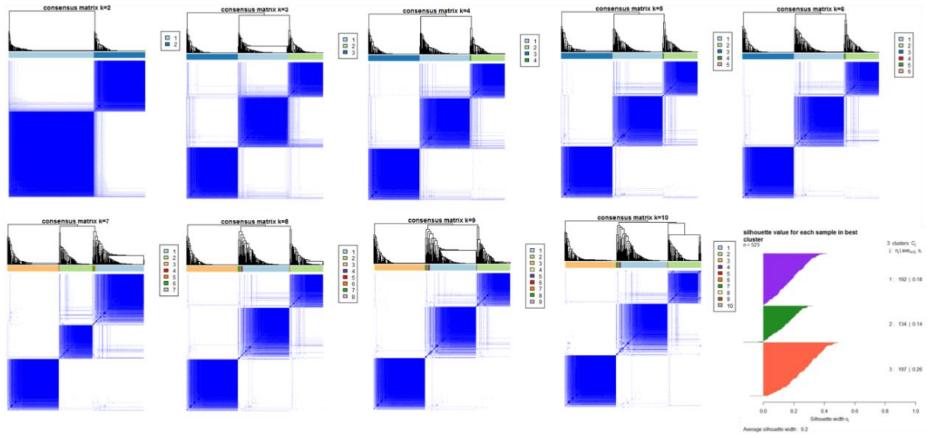

Dataset: TCGA Agilent data
Sample Size: 523 Core samples
Genes used: 500
Subtype identified: 3
Algorithm: Consensus hierarchical clustering using Pearson Correlation
Linkage method: Average

E. **RNA-Seq - TCGA**

(a) Validation of predictive 500 genes set and Identification of three GBM subtypes based on Unsupervised Consensus hierarchical clustering using 168 TCGA RNASEQ data as a validation set. The clustering shown ranging from k=2 to k=10. Silhouette plot for identification of core samples shown for most stable k=3 cluster.

(b) Identification of three GBM subtypes using 150 core samples with predictive 500 genes set. The clustering shown ranging from k=2 to k=9. Silhouette plot for identification of core samples shown for most stable k=3 cluster.

a

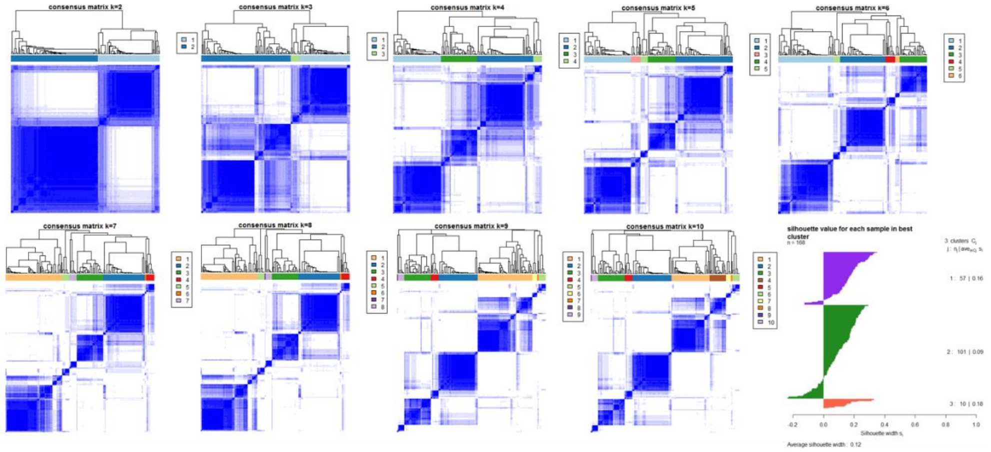

Dataset: TCGA RNASEQ data
Sample Size: 168
Genes used: 500
Subtype identified: 3
Algorithm: Consensus hierarchical clustering using Pearson Correlation
Linkage method: Average

b

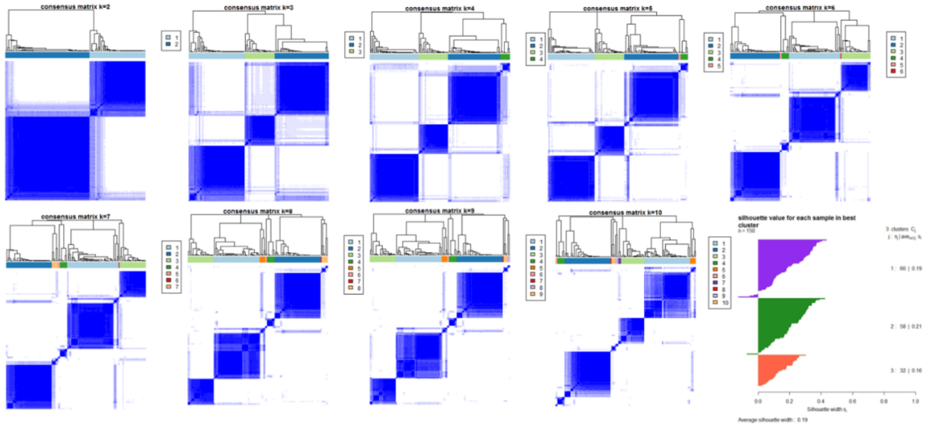

Dataset: TCGA RNASEQ data
Sample Size: 150 Core samples
Genes used: 500
Subtype identified: 3
Algorithm: Consensus hierarchical clustering using Pearson Correlation
Linkage method: Average

F. Using Wang, *et al.*[12] 150 Gene Model

TCGA Affy U133A 496 core samples:
Consensus Hierarchical Clustering using 150 gene-set

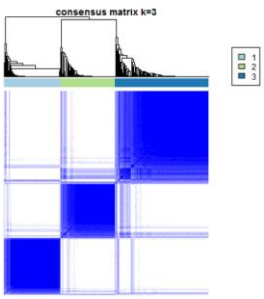

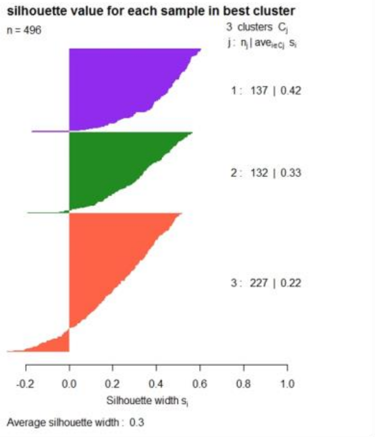

Subtype identity of Cluster 1, 2, 3 above, was based on 150 subtype specific genes from Wang, *et al.* 2017
1- Classical
2-Proneural
3-Mesenchymal

Overlap of Wang, *et al.* 150 gene-set vs our 500 gene-set:
Only 21 genes common between the two genesets.

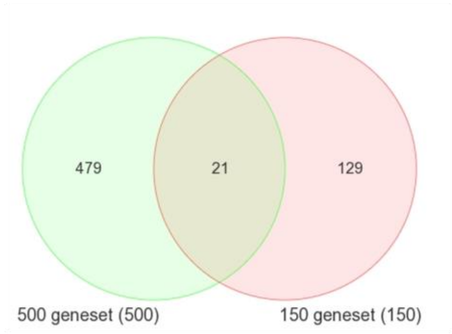

Supplemental 3

A. Comparing 4 GBM subtypes that were previously identified using 840 gene set and the current 3 subtypes identified using the 500 gene set, our analyses demonstrated that original Proneural and Neural subgroups now classified as the same cluster using the 500 gene set, TCGA training set (n_1=496).

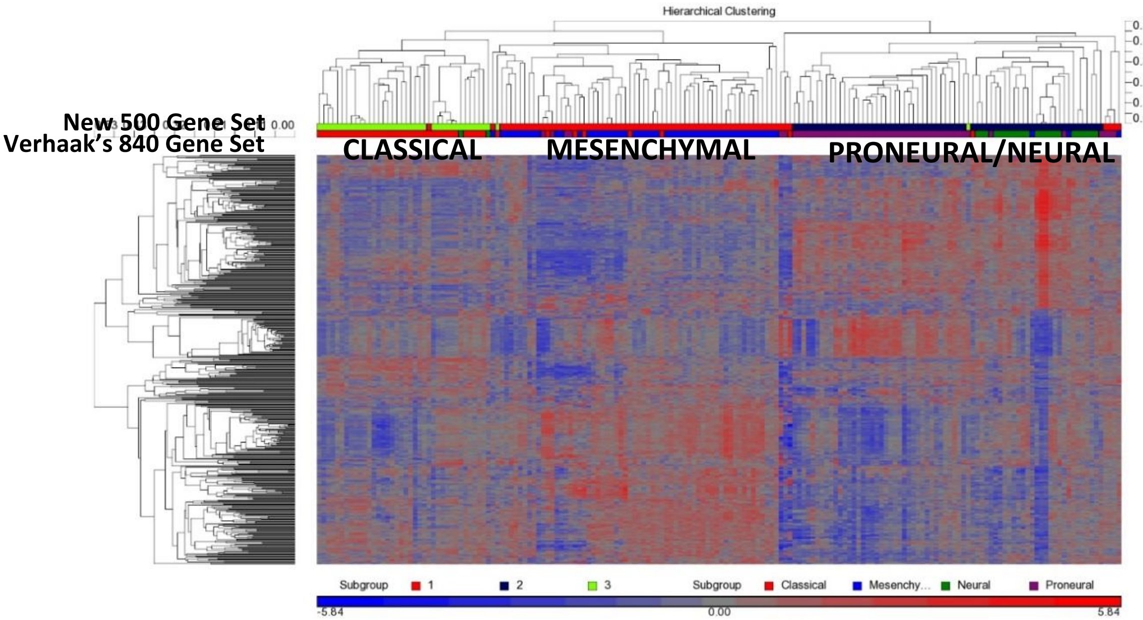

B. Ingenuity pathway analysis (IPA) software was used to identify significant signaling and metabolic pathways. We have performed ANOVA using the three subtypes and have identified subtype-specific genes for each subtype with the cut-off of p-value < 0.05 (Fisher Exact Test) and Fold-change|FC| > 2. We have identified 195 genes specific to Subtype 1 (Mesenchymal), and 156 genes specific to Subtype 2 (Proneural/Neural) and 49 genes specific to Subtype 3 (Classical). We have performed Ingenuity pathway analyses using these subtype specific genes, 195 genes (Subtype 1-Mesenchymal), 156 genes (Subtype 2- Proneural/Neural) and 49 genes (Subtype 3- Classical) to identify top canonical pathways associated with each GBM-subtype. Mesenchymal subtype is enriched in cellular immune response, while Classical subtype and Proneural/Neural subtypes were enriched in neurotransmitter and other nervous system signalling, and intracellular and second messenger signalling as shown below. The significant pathways of three subgroups were shown in Supplemental 4.

Mesenchymal Subtype

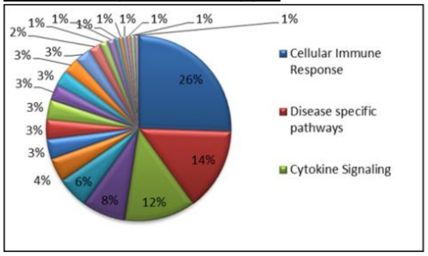

Proneural/Neural Subtype

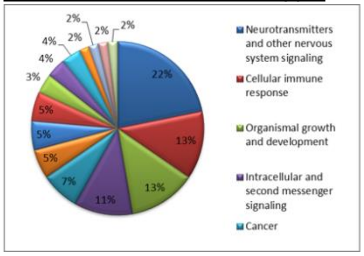

Classical Subtype

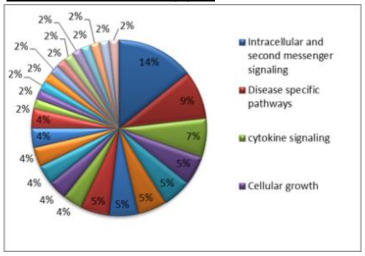

C. Validation using randomly selected 500 genes to perform a consensus cluster analyses of TCGA training set (n_1=496), three subtypes could not be created, supporting the 500-gene-classifier was essential to construct the three GBM-subtypes.

Input: Randomly chosen 500 genes to cluster 496 core samples of Affy-U133A

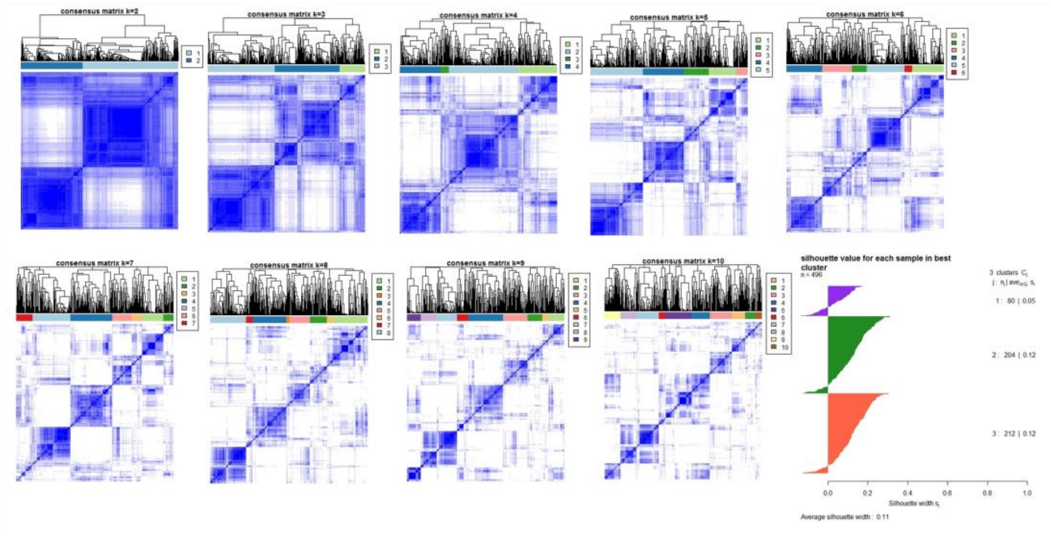

We have also performed the classification by reducing to a minimal gene set of 100-genes with highest MAD, but we did not observe any clustering effect (k=3), there were no 3 clusters observed below.

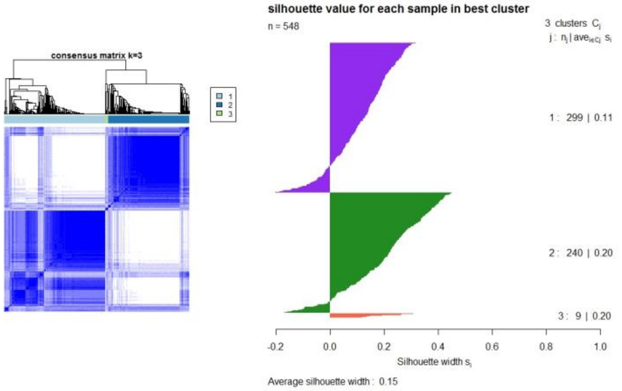

D. Distribution of GBM subtypes among different age groups in TCGA training set (Affymetrix),

D

Age (years)	Subtype	No.	%
**≤18**	Mesenchymal	2	33%
	Proneural/Neural	3	50%
	Classical	1	17%
	**Total**	6	100%
**19-45**	Mesenchymal	29	33%
	Proneural/Neural	51	58%
	Classical	8	9%
	**Total**	88	100%
**>45**	Mesenchymal	184	41%
	Proneural/Neural	156	35%
	Classical	110	24%
	**Total**	450	100%

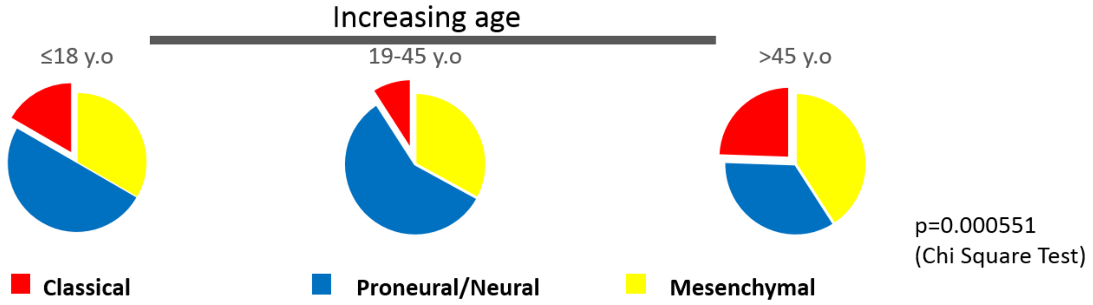

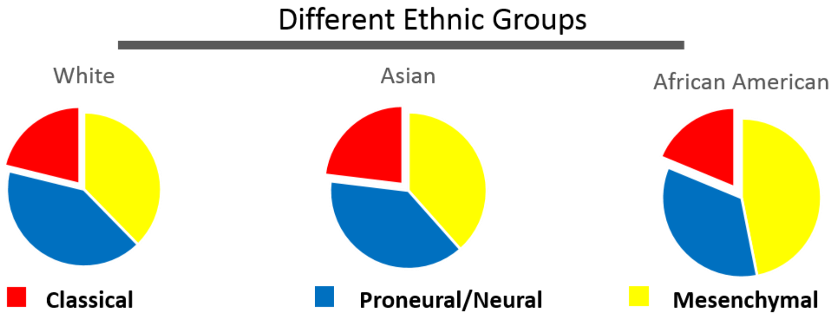

Ethnic Group	Subtype	No.	%
**White**	Mesenchymal	162	37.7%
	Proneural/Neural	177	41.2%
	Classical	91	21.2%
	**Total**	430	100%
**Asian**	Mesenchymal	5	38.5%
	Proneural/Neural	5	38.5%
	Classical	3	23.1%
	**Total**	13	100%
**African American**	Mesenchymal	15	46.9%
	Proneural/Neural	11	34.4%
	Classical	6	18.8%
	**Total**	32	100%

*21 tumors (11 Mesenchymal, 7 Proneural/Neural, 3 Classical do not have clinical information on ethnicity available.

E. **Relevance of 500 Signature Gene Set Classifier among Asian Adult GBMs.** Heatmap showing 3 GBM subtypes among 51 core samples in a cohort of 61 Asian patients (58 patients from GSE42670 and 3 new patients from the same institution). The 500 gene classifier was able to recapitulate three GBM subtypes in a cohort of Asian patients with GBM. Consensus clustering matrix for k=3 of 51 core samples in Asian adult GBM cohort. Silhouette plot identified 51 core samples. Consensus clustering CDF for k=2 to k=10.

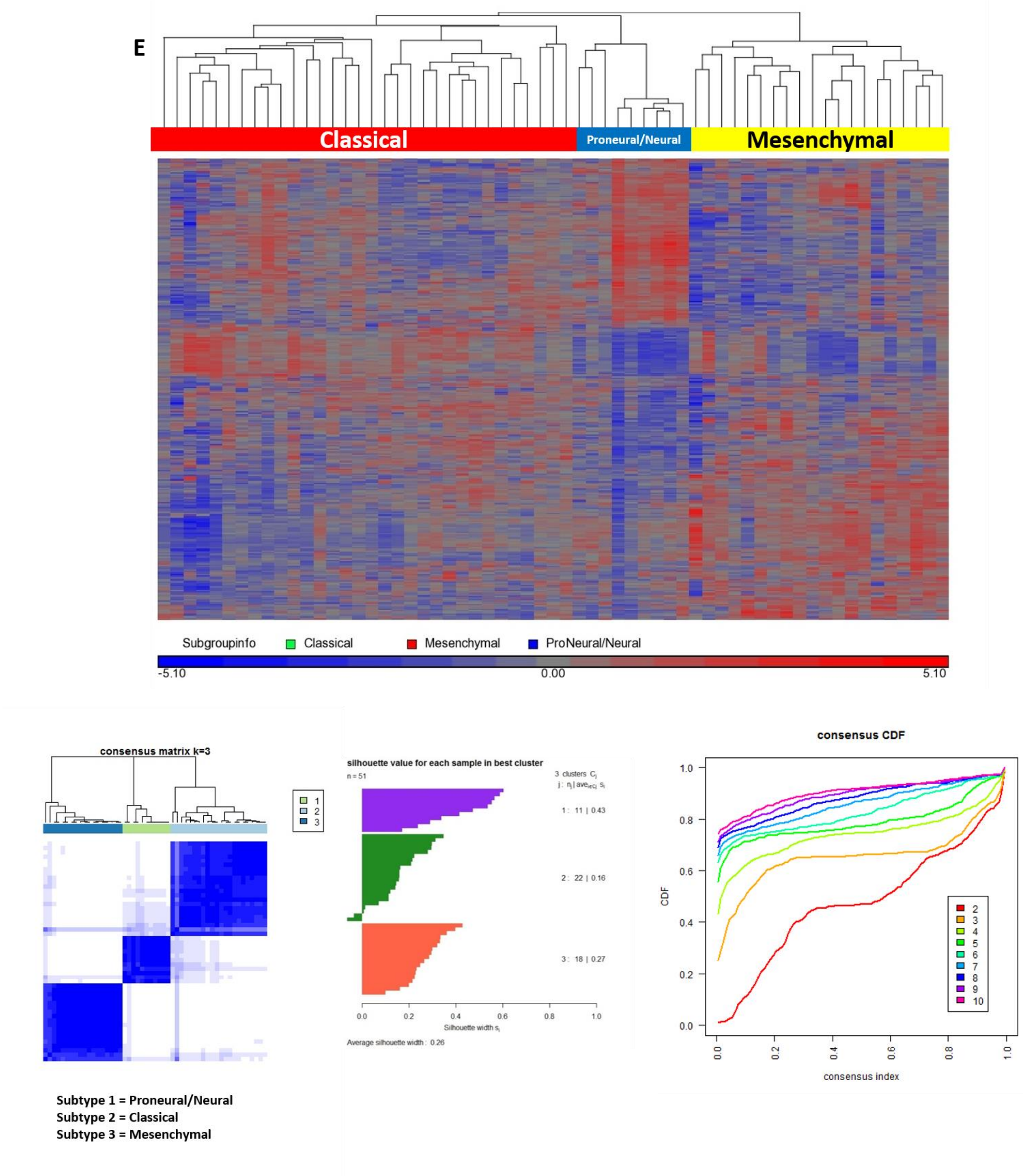

F. 25 Caucasian pediatric GBMs. The 500 gene classifier was able to recapitulate 3 GBM subtypes in Asian adult patient GBM cohort but not pediatric GBMs in a Caucasian-predominant childhood cohort.

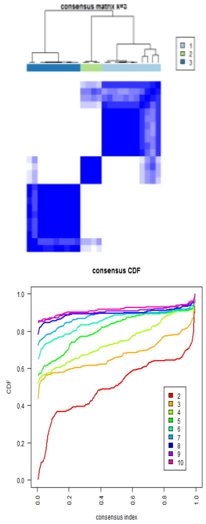

G. Comparison of subtype patterns across all patient populations of Caucasians, Koreans and Chinese did not reveal a consistent statistical trend, likely due to the larger TCGA Caucasian cohorts compared to smaller Asian cohorts and demographics differences.

Group	Proneural/Neural	Mesenchymal	Classical	Chi-sq (p-value)	Remarks
**Caucasian-1 (Affymetrix)**	200	191	104		
**Asian Cohort 1**	11	18	22	0.000831	p < .05 significant
**Caucasian-2 (Agilent)**	197	192	134		
**Asian Cohort 2**	15	17	13	0.821	p > .05 Not significant
**Caucasian-3 (RNA-Seq)**	60	58	32		
**Asian Cohort 3**	22	23	14	0.908	p > .05 Not significant
**Caucasian-1 (Affymetrix)**	200	191	104		
**Asian Cohort 2**	15	17	13	0.425	p > .05 Not significant
**Caucasian-2 (Agilent)**	197	192	134		
**Asian Cohort 3**	22	23	14	0.927	p > .05 Not significant
**Caucasian-3 (RNA-Seq)**	60	58	32		
**Asian Cohort 1**	11	18	22	0.005	p < .05 significant
**Caucasian-1 (Affymetrix)**	200	191	104		
**Asian Cohort 3**	22	23	14	0.855	p > .05 Not significant
**Caucasian-2 (Agilent)**	197	192	134		
**Asian Cohort 1**	11	18	22	0.013	p < .05 significant
**Caucasian-3 (RNA-Seq)**	60	58	32		
**Asian Cohort 2**	15	17	13	0.531	p > .05 Not significant

H. Schematic

Graphical Abstract

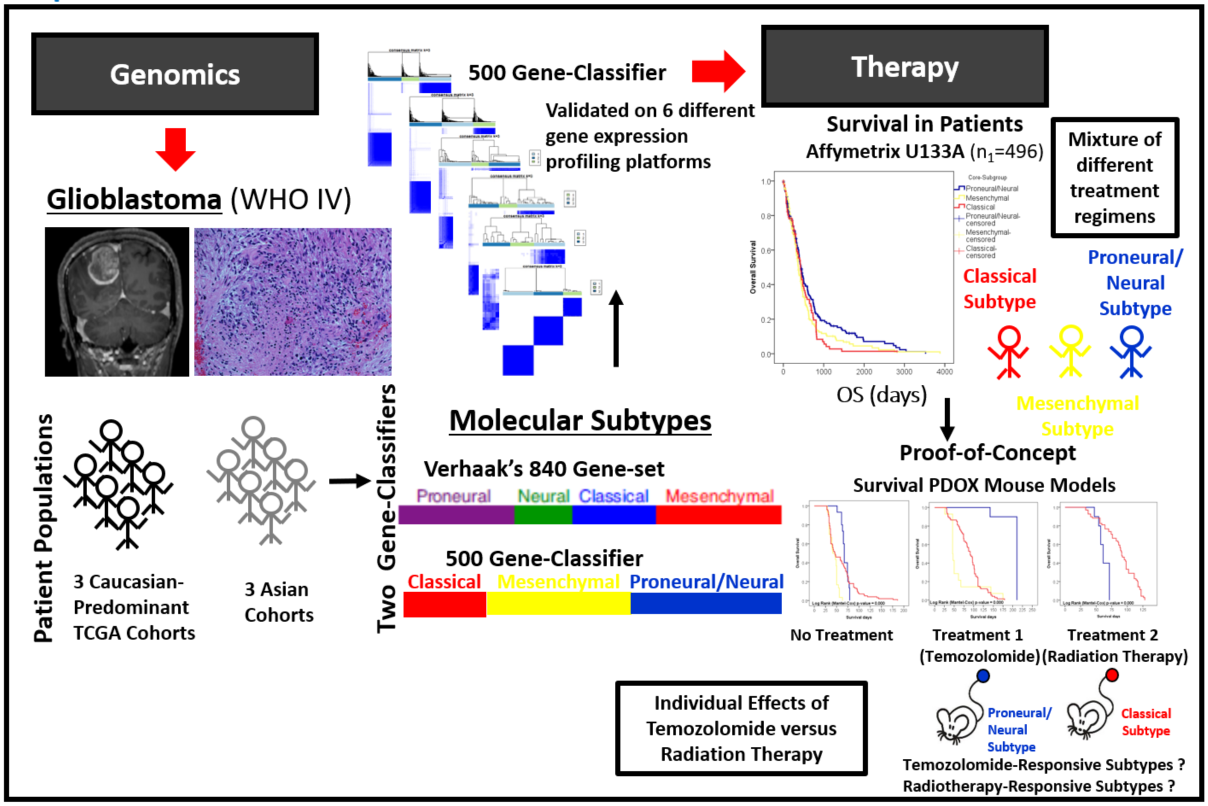

Supplementary Tables

Supplemental 4

Identification of subtype specific pathways using IPA: List of differentially expressed genes between three GBM subtypes identified using ANOVA with P-value < 0.05 and |FC| > 2 shown in Gene List 4 below were input into IPA to derive significant pathways (Supplementary Table A-C).

Supplemental 5

Average number of mice per patient-derived orthotopic (PDX) model for GBM derived from patients in Asian Cohort 1, in No Treatment, Treatment 1 (Temozolomide) and Treatment 2 (Radiation Therapy) groups of mice.

No treatment		Subtype	Number of animals
1	PDX1	Classical	7
2	PDX11	Classical	9
3	PDX12	Classical	10
4	PDX14	Classical	12
5	PDX2	Classical	14
6	PDX3	Classical	9
7	PDX4	Classical	12

8	PDX5	Classical	9
9	PDX6	Classical	8
10	PDX8	Classical	10
11	PDX10	Mesenchymal	18
12	PDX9	Mesenchymal	12
13	PDX7	ProNeural/Neural	15
	**Total**		145
	**Average**		11
**Treatment 1 (Temozolomide)**			
1	PDX1	Classical	8
2	PDX11	Classical	10
3	PDX12	Classical	9
4	PDX14	Classical	4
5	PDX2	Classical	8
6	PDX3	Classical	7
7	PDX4	Classical	7
8	PDX5	Classical	10
9	PDX8	Classical	10
10	PDX10	Mesenchymal	10
11	PDX9	Mesenchymal	4
12	PDX7	ProNeural/Neural	10
	**Total**		97
	**Average**		8
**Treatment 2 (Radiation Therapy)**			
1	PDX1	Classical	8
2	PDX11	Classical	9
3	PDX12	Classical	10
4	PDX2	Classical	15
5	PDX3	Classical	9
6	PDX4	Classical	8
7	PDX5	Classical	10
8	PDX7	ProNeural/Neural	10
	**Total**		79
	**Average**		10

Gene Lists

Gene List A:

500-Gene Classifier for three GBM subtypes

Gene List B:

List of 1500 differentially expressed gene set that were overlapped between all three datasets of TCGA GBM samples

Gene List C:

Identification of subtype specific genes: List of differentially expressed genes between three GBM subtypes (Supplemental 4A-C) identified using ANOVA with P-value < 0.05 and $|FC| > 2$.
